# Pancreatic cyst fluid analysis for differential diagnosis between benign and malignant lesions

**DOI:** 10.3892/ol.2012.1071

**Published:** 2012-12-12

**Authors:** RENATA TALAR-WOJNAROWSKA, MAREK PAZUREK, LUKASZ DURKO, MALGORZATA DEGOWSKA, GRAZYNA RYDZEWSKA, JACEK SMIGIELSKI, ADAM JANIAK, MAREK OLAKOWSKI, PAWEŁ LAMPE, PIOTR GRZELAK, LUDOMIR STEFANCZYK, EWA MALECKA-PANAS

**Affiliations:** 1 Department of Digestive Tract Diseases, Medical University, Łódź 90-153;; 2 Department of Gastroenterology, Central Clinical Hospital of the Ministry of Interior and Administration, Warsaw 02-507;; 3 Department of Thoracic Surgery, General and Oncological Surgery, Medical University, Łódź 90-549;; 4 Department of Gastroenterological, Oncological and General Surgery, Medical University, Łódź 90-153;; 5 Department of Surgery of Digestive Tract Diseases, Silesian Medical University, Katowice 40-752;; 6 Department of Radiology, Medical University, Łódź 90-153, Poland

**Keywords:** pancreatic cyst, fluid analysis, carcinoembryonic antigen, CA19-9, amylase

## Abstract

The majority of pancreatic cysts are detected incidentally when abdominal imaging is performed during unrelated procedures. The aim of the present study was to assess the diagnostic utility and clinical value of carcinoembryonic antigen (CEA), carbohydrate antigen 19-9 (CA 19-9) and amylase analysis in pancreatic cyst fluid. The study included 52 patients with pancreatic cystic lesions, who underwent fine-needle aspiration biopsy to collect cystic fluid for cytological and biochemical analysis. Cysts were classified as benign (simple cysts, pseudocysts and serous cystadenomas) in 36 patients or premalignant/malignant (mucinous cyst-adenomas, intraductal papillary mucinous neoplasm and cystadenocarcinomas) in 16 patients. CEA and CA 19-9 were elevated in patients with malignant cysts (238±12.5 ng/ml and 222±31.5 U/ml, respectively) compared with benign lesions (34.5±3.7 ng/ml and 18.5±1.9 U/ml, respectively; P<0.001). Based on these results, the sensitivity and specificity of CEA were 91.8 and 63.9% and of CA 19-9 were 81.3 and 69.4%, respectively. Mean amylase levels in benign lesions (27825.7±91.9 U/l) were higher compared with malignant pancreatic cysts (8359.2±32.7 U/l; P<0.05). Cyst fluid analysis may prove a safe and useful adjunct for the differential diagnosis of pancreatic cystic lesions. In the present study, promising results for CEA and CA 19-9 have been demonstrated, however, the clinical value of these molecules must be confirmed.

## Introduction

Pancreatic cysts are commonly detected incidentally in patients undergoing abdominal imaging for unrelated procedures. Simple (retention) cysts, pseudocysts and serous cystadenomas lack malignant potential. However, mucinous cystic neoplasms and intraductal papillary mucinous neoplasm (IPMN) have a malignant potential and require surgical treatment (1–4). Due to the possibility of malignancy in specific pancreatic cysts, it is important differentiate between benign and malignant lesions to determine whether surgical resection or conservative management is required.

Analysis of cystic fluid may be useful for distinguishing between benign and malignant pancreatic lesions. To date, several tests using cyst fluid to diagnose premalignant cyst are in use. These include cytology, tumor markers [i.e., carcinoembryonic antigen (CEA) or carbohydrate antigen 19-9 (CA 19-9)], biochemical markers (i.e., amylase) and cyst fluid viscosity. Among these tests, CEA has the highest diagnostic accuracy for discriminating premalignant mucinous from nonmucinous cysts (5,6). However, CEA cannot differentiate between a premalignant cyst and a malignant lesion (7,8).

CA 19-9 is the most popular serum-based marker for pancreatic cancer diagnosis and is important for the detection of recurrent disease and surveillance of patients following surgery. Previous studies have demonstrated that CA 19-9 cyst fluid analysis may also be useful for differential diagnosis of pancreatic cysts, particularly in pancreatic cystadenocarcinoma detection (9–11). Current data are insufficient to reliably determine the clinical value of CA 19-9 cyst fluid analysis, however, if a panel of tests is performed in conjunction with clinical and radiological observations, the identity of pancreatic cysts is predicted with a high degree of reliability.

The third analyzed parameter, pancreatic cyst fluid amylase, may be particularly useful for the identification of pseudocysts. Distinguishing pseudocysts from malignant cystic tumors is essential during selection of appropriate surgical procedures. Pseudocysts may be managed by observation or, in specific cases with endoscopic or surgical drainage. The amylase content of pseudocysts is almost always high, whereas the level in neoplastic cysts is generally low. However, cystic tumors of all types may exhibit elevated amylase levels. Consequently, the efficacy of amylase measurements in pancreatic cyst fluids is limited, although low values indicate a neoplastic tumor (6,8,12,13).

The aim of the present study was to assess the diagnostic utility and clinical value of CEA, CA 19-9 and amylase analysis in pancreatic cyst fluid.

## Materials and methods

### Sample collection and classification

The present study included 52 patients (28 males and 24 females) with pancreatic cystic lesions. Patients underwent fine-needle aspiration biopsy to collect cystic fluid for cytological and biochemical analysis. Informed consent was obtained from all patients. The study was approved by the ethical committee of Lodz Medical University. Based on surgical histopathology, cytology results and/or imaging follow-up (>18 months), cysts were classified as benign (simple cysts, pseudocysts and serous cystadenomas) or premalignant/malignant (mucinous cystadenomas, IPMNs and cystadenocarcinomas) in 36 and 16 patients, respectively.

### Pancreatic cyst analysis

The following characteristics were analyzed: maximum pancreatic cyst diameter, cyst number and location, wall thickness, mural nodules, pancreatic duct communication and/or dilation and presence of septations or calcifications. Following cyst fluid aspiration, a portion of the specimen was sent to the chemistry laboratory of the Department of Digestive Tract Diseases for CEA, CA 19-9 and amylase analysis. The fluid was also examined by a cytopathologist.

### Analysis of patient characteristics

Age and gender of patients, presenting symptoms and medical history of acute or chronic pancreatitis were also assessed. Criteria for resection included premalignant/malignant lesions identified by cytology or fluid analysis and suspicious radiographical observations, including cyst size >3 cm and intramural nodules, pancreatic duct dilation, peripheral calcifications or associated mass. Resection was also advised for symptomatic benign pancreatic cysts. All patients with premalignant/malignant lesions underwent surgical treatment. Fifteen patients with a final diagnosis of benign lesions also underwent surgical resection due to symptoms or suspicious features of imaging and/or cyst fluid analysis.

### Statistical analysis

Statistical analysis comprised arithmetical mean, median and standard deviation. Mann-Whitney or Fisher’s exact tests were performed to determine differences between groups. P<0.05 was considered to indicate a statistically significant difference. A receiver operating characteristic (ROC) curve depicting the ability to discriminate between benign and premalignant/malignant cysts was plotted for CEA, CA 19-9 and amylase and optimal cut-off points were estimated.

## Results

### Patient characteristics

The mean age of patients included in the present study was 55±3.2 years; there were 28 male (53.8%) and 24 female (46.2%) subjects. A total of 36 cysts were classified as benign and 16 patients had premalignant/malignant cysts (8 mucinous cystadenomas, 4 IPMNs and 4 cystadenocarcinomas). The majority of patients were asymptomatic, whereas 23 patients (14 benign cyst and 9 premalignant/malignant lesions) presented abdominal pain, weight loss and/or jaundice. Nine patients had a previous history of acute pancreatitis, whereas chronic pancreatitis was confirmed in 11 patients.

### Pancreatic cyst characteristics

The mean diameter of pancreatic cyst was 3.3 cm (range, 1.5–8.1 cm). No statistically significant difference was identified between benign and malignant cyst size (3.9±1.8 vs. 3.2±1.2 cm; P>0.05). Cyst localization was identified in the pancreatic head and body or tail of pancreas in 29 (55.8%) and 23 (44.2%) patients, respectively. Cytology was assessed in all patients and was reported as acellular, benign or atypical in 49 patients and positive for malignant cells in 3 patients.

### CEA and CA 19-9 levels

**Figure 1.**
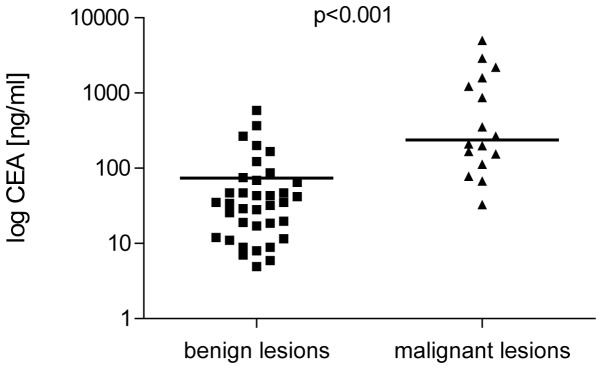
Comparison of cyst fluid CEA levels in patients with benign and malignant pancreatic lesions (results presented in logarithmic scale). CEA, carcinoembryonic antigen.

**Figure 2.**
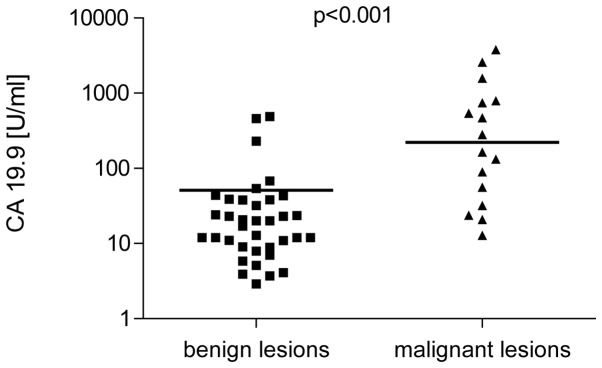
Comparison of cyst fluid CA19-9 levels in patients with benign and malignant pancreatic lesions (results presented in logarithmic scale). CA 19.9, carbohydrate antigen 19-9.

CEA and CA 19-9 were higher in patients with malignant cysts (238±12.5 ng/ml and 222±31.5 U/ml, respectively) compared with benign lesions (34.5±3.7 ng/ml and 18.5±1.9 U/ml; P<0.001; Figs. 1 and 2). Sensitivity and specificity for CEA (cut-off, 45 ng/ml) was 91.8 and 63.9% and for CA 19-9 (cut-off, 37 U/ml) was 81.3 and 69.4%, respectively. Positive predictive value (PPV) of CEA was 53.6% and the negative predictive value (NPV) was 95.8% NPV and PPV of CA 19-9 were 54.2 and 89.3% respectively.

### Amyase levels

**Figure 3.**
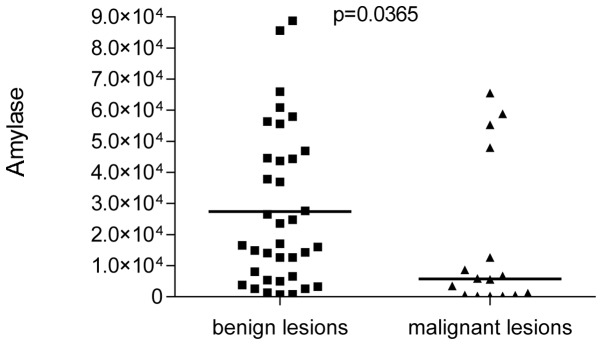
Comparison of cyst fluid amylase levels in patients with benign and malignant pancreatic lesions.

Mean amylase level in benign lesions (27,825.7±91.9 U/l) was identified as significantly higher compared with malignant pancreatic cysts (8,359.2±32.7 U/l; P<0.05; Fig. 3). The highest levels of amylase were observed in pseudocysts (41,778±131.5 U/l). However, the amylase sensitivity and specificity for diagnosis of premalignant/malignant lesions was lower than those of CEA and CA 19-9 (62.5 and 69.4%, respectively). PPV and NPV of amylase was also lower, 47.6 and 80.6%, respectively.

### ROC curve

**Figure 4.**
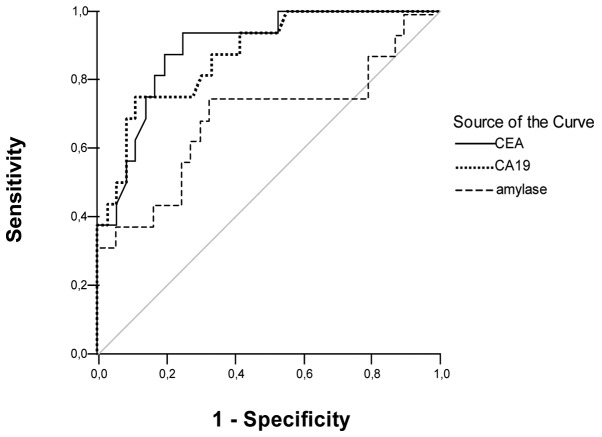
ROC curve analyses of CEA, CA19-9 and amylase fluid levels for the diagnosis of premalignant/malignant pancreatic cysts. CEA, carcinoembryonic antigen; CA 19.9, carbohydrate antigen 19-9; ROC, receiver operating characteristic.

ROC curve for the abilities of CEA, CA 19-9 and amylase to distinguish between benign and malignant lesions is plotted in Fig. 4. Area under the curve was 0.892 [95% confidence interval (CI), 0.803–0.981] for CEA, 0.873 (95% CI, 0.773–0.973) for CA 19-9 and 0.684 (95% CI, 0.508–0.861) for amylase.

## Discussion

Pancreatic cysts are a heterogenous tumor group with varied clinical presentation and malignant potential. Distinguishing between benign inflammatory or serous lesions from potentially malignant mucinous cystic tumors is vital for clinical differential diagnosis of pancreatic cysts. Aspiration of pancreatic fluid cysts for additional markers has been hypothesized to be important for patient management.

The present study identified that the median cyst fluid CEA and CA 19-9 levels in premalignant/malignant cysts was significantly higher than in benign cysts (P<0.001). Sensitivity for CEA and CA 19-9 was 91.8 and 81.3%, respectively, for mucinous lesions. Previously, the combination of CEA fluid assessment and K-ras mutation analysis levels was confirmed to maximize the diagnostic yield of pancreatic cyst biopsy and improve sensitivity and specificity of cyst classification (14). However, the current cost of DNA mutational testing limits the availablity of this analysis in specific laboratories.

To improve the efficacy of pancreatic cyst diagnosis, additional tumor markers have been investigated, including CA 19-9 and amylase. Analysis of CA 19-9 fluid levels for the differential diagnosis of pancreatic cysts is controversial. CA 19-9 fluid levels are currently considered to be less specific compared with CEA, particularly for detection of mucinous cysts (15,16). However, a study performed by Wu *et al* identified that CA 19-9 fluid assessment had higher sensitivity and specificity compared with CEA for detection of pancreatic cystadenocarcinomas (83.3 and 94.4 vs. 61.1 and 92.2%, respectively) (9). Therefore, we hypothesized that the combination of analyzed markers may improve their accuracy for the differential diagnosis of pancreatic cysts.

A previous study demonstrated that the sensitivity of cyst fluid CEA combined with CA 19-9 measurement was higher than single tumor marker examination (9). By contrast, Brugge *et al* performed analysis of pancreatic cyst fluid in a large group of patients and concluded that fluid CEA alone is most useful for diagnosis of malignant pancreatic cysts. The combination of additional tests, including CA 19-9 as well as CA 72-4, CA 125 and CA 15-3, was not identified to be more accurate. Moreover, the addition of cyst morphology or cytology to the CEA value did not improve diagnostic accuracy (16).

In the present study, CA 19-9 levels, with a cut-off value of 37 U/ml, were elevated in patients with malignant cysts compared with benign lesions. Results are consistent with previous studies reporting that low CA 19-9 fluid levels (less than 37 U/ml) suggest benign lesions (13,17). The CA 19-9 cut-off value is most frequently utilized (9,10,17). Increasing the cut-off value for CA 19-9 to support the diagnosis of a malignant cyst has been previously demonstrated to increase the specificity but decrease the sensitivity of the test. Frossard *et al* reported that a CA 19-9 value greater than 50,000 U/ml in the cyst fluid had an 86% sensitivity and 85% specificity for distinguishing cystadenocarcinoma from other cystic lesions. However, this high cut-off value had a sensitivity of only 15% for detection of mucinous cysts. The authors concluded that this high threshold for CA 19-9 is suitable for the detection of malignancies but is insensitive for premalignant lesions (15).

In the present study, sensitivity of the third analyzed parameter, amylase, was 62.5% and the specificity was 69.4%, which was lower than those of CEA and CA 19-9. Previous studies on the clinical efficacy of amylase for differential diagnosis of pancreatic cysts are inconsistent. However, the parameter may be useful for confirmation of pseudocyst diagnosis, particularly in patients with a medical history of pancreatitis. Snozek *et al* reported that CEA and amylase fluid levels less than 30 ng/ml and more than 8500 U/l, respectively, were observed in 91% of pseudocysts (12). In addition, Attasaranya *et al* demonstrated that the median level of amylase was higher in pseudocysts compared with all other cystic lesions (19,834 vs. 882 U/l, respectively), however, this difference was identified to be at the limit of statistical significance (P=0.05). The authors reported a high sensitivity (100%) and specificity (63.6%) of cyst fluid amylase at a cut-off of 5,000 U/l for differentiating pseudocysts from all other pancreatic cysts (8).

Increased amylase fluid levels are not specific for pseudocysts and has been observed in additional cysts, including mucinous cystadenomas and IPMN (6,18,19). Le Borgne *et al,* following surgical resection of 398 pancreatic cystic tumors, observed that 6% of mucinous cystadenomas and 10% of cyst-adenocarcinomas were associated with pancreatic ducts (19). Park *et al* identified that 54% of noninflammatory cysts, including mucinous cystic neoplasms had an increased level of amylase. However, lower amylase levels were identified in malignant mucinous cysts than benign mucinous cysts (6).

At present, the efficacy of amylase level analysis for the differentiation of benign from premalignant/malignant cysts has not been determined. However analysis of amylase may be useful for patients with a medical history of pancreatitis where there is a greater probability of a pseudocyst. Further evaluation of this efficacy, based on long-term prospective studies in patients with pancreatic cysts, must be performed.

In conclusion, the present study indicates that analysis of pancreatic cyst fluid may be a safe and useful adjunct for the differential diagnosis of pancreatic cystic lesions. This analysis may distinguish inflammatory and benign neoplastic cysts from premalignant/malignant pancreatic lesions. Results appear promising, not only for CEA, but also for CA 19-9, however, the clinical value of these markers must be confirmed.
